# Editorial: Environmental challenges from global warming/climate change, urbanization and pollution to wild birds and poultry

**DOI:** 10.3389/fphys.2026.1900269

**Published:** 2026-06-25

**Authors:** Frédéric Angelier, Gregoy Y. Bedecarrats, Vincent M. Cassone, Pierre J. Deviche, Israel Rozenboim, Devraj Singh, Karen L. Sweazea, Colin G. Scanes

**Affiliations:** 1 Centre d’etudes biologiques de Chizé (CEBC), UMR7372, CNRS-La Rochelle Université, Villiers-en-Bois, France; 2 Department of Animal Bioscience, University of Guelph, Guelph, ON, Canada; 3 Department of Biology, University of Kentucky, Lexington, KY, United States; 4 School of Life Sciences, Arizona State University, Tempe, AZ, United States; 5 The Robert H. Smith Faculty of Agriculture, Food and Environment Hebrew University of Jerusalem, Rehovot, Israel; 6 College of Health Solutions, Arizona State University, Phoenix, AZ, United States; 7 Department of Biological Sciences, University of Wisconsin Milwaukee, Milwaukee, WI, United States

**Keywords:** birds, climate change, global warming, pollution, urbanization

Both wild birds and poultry can be negatively impacted by human activities with two of the worse culprits being anthropogenic global warming/climate change and urbanization, which are the focus of this special topic in *Frontiers in Physiology*.

Globally, climate has changed over the past 100 years due to industrial and other human activities with increased release of greenhouse gases (GHG) such as carbon dioxide, methane and nitrous oxide into the atmosphere. While atmospheric concentrations of carbon dioxide have oscillated from 250 to 180 ppm every 50,000 years for the past 800,000 years, they have rapidly risen to 423 ppm beginning with the industrial revolution and more so since 1960 (NOAA, 2025; NASA, 2026). As a consequence, average global temperatures have rapidly risen by 1.5°C (Intergovernmental Panel on Climate Change (IPCC), 2023). Global climate change affects breeding cycles, migratory patterns, food availability, and multiple aspects of avian physiology.

An obvious corollary to global warming/climate change are increases in the incidence of high temperatures in affecting wild birds and poultry. There are multiple adverse effects of heat stress on avian physiology. For instance, plasma concentrations of the stress hormone, corticosterone, are elevated in heat stressed chickens (Rezar et al.). Moreover, intestinal functioning is reportedly shifted in heat stressed chickens (Liu et al.). Duodenal villus height and, consequently area for absorption of nutrients, was reduced by heat stress in chickens selected for either low- and high-water-efficiency. Moreover, expression of gut barrier mRNA and proteins that assure gut integrity were increased including the following:

1. Barrier-forming claudins - CLDN1, CLDN5, CLDN8, CLDN16, and CLDN22.
2. Tight junction proteins - PALS1-associated tight junction protein (PATJ) and gap junction proteins alpha 1 (GJA1) and alpha 3 (GJA3).
3. Cadherens junction proteins - Cadherin 2 (CDH2) and catenin alpha 2 (CTNNA2).

Liu et al. Similarly, heat stress reduced body mass and induced marked shifts in the expression of multiple genes associated with differentiation, development, as well as lipid accumulation in pectoralis muscles of turkey poults raised at 39°C versus 35°C (Reed et al.).

Sulaiman et al. also examined the effects of temperature on the expression of genes related to fat metabolism during embryonic development of chicks incubated at elevated temperatures (heat conditioned) or under control conditions. Post hatching, the subcutaneous adipose tissue from heat conditioned chick embryos exhibited decreased expression of the following genes: CCAAT/enhancer-binding protein alpha (C/EBPα), diacylglycerol O-acyltransferase 2 (DGAT2), peroxisome proliferator-activated receptor gamma (PPARγ), sterol regulatory element-binding protein 1 (SBEBP1), and neuropeptide Y (NPY). In contrast, expression of the hormone sensitive lipase (HSL) gene in adipose tissue is increased. These results demonstrated that heat and increased temperatures can have important impacts on fat metabolism in poultry.

Although an increase in heat shock proteins (HSP) in response to heat stress/increased temperatures was reported in some bird species, this appears not to be universal. For instance, hypothalamic expression of HSP90b1 is reportedly increased in chickens subjected to heat stress for 12 or 24 hours (Wan et al., 2017). However, heat stress does not affect blood expression of either constitutive heat shock protein (HSP70) or inducible HSP90 in zebra finch (*Taeniopygia guttata*) (Finger et al., 2018) or HSP70 as observed in chickens (Rezar et al.). In wild nestling tree swallows (*Tachycineta bicolor*) subjected to heat stress (Woodruff et al., 2025), expression of HSP90AA1 in blood does not track with the recent heat index making it an unsatisfactory biomarker (Woodruff et al.).

Phenology provides evidence that animal and plant life are adapting to global warming. Guillemette et al. examined whether sea surface temperature, air temperature, and/or photoperiod are responsible for the seasonal variation in egg laying and core body temperature in common eiders (*Somateria mollissima mollissima*). There were progressive increases in the core body temperature in the 40 days preceding egg laying, and importantly, there were strong relationships between core body temperatures and sea surface temperature, air temperature, and photoperiod during that pre-laying period.

Bird populations are negatively impacted by the environmental challenges of urbanization resulting in loss and degradation of habitat due to the artificialization of the environment as well as chemical pollution (air and water). These challenges can contribute to reduced reproductive success and increases in both morbidity and mortality. Lane et al. reviewed acute effects of stressors elevating plasma concentrations of corticosterone in wild birds. This was a prelude to examining putative differences in plasma concentrations of corticosterone in female song sparrows (*Melospiza melodia*) in an urban as compared to a rural environment. Basal plasma concentrations of corticosterone were depressed in urban song sparrows. Moreover, the increase in plasma concentrations of corticosterone in song sparrows subjected to restraint stress was smaller in urban than rural song sparrows. This is consistent with the chronic stress of urban life down-regulating the hypothalamic-pituitary-adrenocortical axis (HPA).

According to the United States Environmental Protection Agency (EPA) (2025), ground level ozone (O_3_) is a “*harmful pollutant*” and the main component of “*smog*” with adverse effects on human health, particularly in children and the elderly. It is formed in the atmosphere by chemical reactions between oxides of nitrogen (NO_x_) formed from human activities and volatile organic compounds (United States Environmental Protection Agency, 2025). American robins (*Turdus migratorius*) reportedly exhibit increased circulating concentrations of corticosterone as ambient atmospheric concentrations of nitric dioxide (NO_2_) rise (Abolins-Abols et al.). Exposure to ozone is accompanied by decreased total glutathione concentrations in erythrocytes from zebra finches (Ziegler et al.). Moreover, there is an increase in the ratio of reduced (GSH) to oxidized (GSSG) glutathione [GSH/GSSG ratio] in red blood cells of captive zebra finches on a ω3-rich diet subjected to heat stress (Ziegler et al.). In a similar manner, there were increases in circulating concentrations of corticosterone in Japanese quail (*Coturnix coturnix japonica*) and American kestrels (*Falco sparverius*) exposed to benzene, toluene, NO_2_, and sulfur dioxide (SO_2_) (Cruz-Martinez et al., 2015). All these studies support the idea that exposure to such pollutants may harm wild birds through detrimental effects on their physiology.

The internal cellular clock is thought to have first developed 2–3 billion years ago (Dvornyk et al., 2003). The circadian oscillator is entrained by environmental zeitgebers. To date, much research has focused exclusively on the development of an internal cellular clock in laboratory rodents, zebrafish (*Danio rerio*), and the fruit fly (*Drosophila melanogaster*) (Vallone et al., 2007). In a seminal article, Wellard et al. review the role of the internal cellular clock with “*rhythmic gene expression*” in the chick embryo and its importance in ensuring regulated development.

A stressor unique to urban areas is artificial light at night. Reid et al. examined the effects of artificial light at night on captive adult zebra finches demonstrating alterations in the circadian rhythms of plasma concentrations of glucose and telomere length, but not oxidative status. In contrast to heat stressed chickens (Rezar et al.), there is a lack of correlation between concentrations of corticosterone in feathers and the degree of urbanization in southern lapwing (*Vanellus chilensis*) in Chile (Quirici et al.).

Collectively, the studies published in this Research Topic underscore the multifaceted ways in which anthropogenic climate change and urbanization challenge avian physiology across life stages, species, and ecological contexts.
